# Commissioning and early experience with a new-generation low-energy linear accelerator with advanced delivery and imaging functionalities

**DOI:** 10.1186/1748-717X-6-129

**Published:** 2011-09-30

**Authors:** Alessandro Clivio, Giorgia Nicolini, Eugenio Vanetti, Antonella Fogliata, Luca Cozzi

**Affiliations:** 1 Medical Physics Unit, Oncology Institute of Southern Switzerland, Bellinzona, Switzerland

**Keywords:** UNIQUE linear accelerator, RapidArc, Beam Commissioning

## Abstract

**Background:**

A new-generation low-energy linear accelerator (UNIQUE) was introduced in the clinical arena during 2009 by Varian Medical Systems. The world's first UNIQUE was installed at Oncology Institute of Southern Switzerland and put into clinical operation in June 2010. The aim of the present contribution was to report experience about its commissioning and first year results from clinical operation.

**Methods:**

Commissioning data, beam characteristics and the modeling into the treatment planning system were summarized. Imaging system of UNIQUE included a 2D-2D matching capability and tests were performed to identify system repositioning capability. Finally, since the system is capable of delivering volumetric modulated arc therapy with RapidArc, a summary of the tests performed for such modality to assess its performance in preclinical settings and during clinical usage was included.

**Results:**

Isocenter virtual diameter was measured as less than 0.2 mm. Observed accuracy of isocenter determination and repositioning for 2D-2D matching procedures in image guidance was <1.2 mm. Concerning reproducibility and stability over a period of 1 year, deviations from reference were found <0.3 ± 0.2% for linac output, <0.1% for homogeneity, similarly to symmetry. Rotational accuracy of the entire gantry-portal imager system showed a maximum deviation from nominal 0.0 of <1.2 mm. Pre treatment quality assurance of RapidArc plans resulted with a Gamma Agreement Index (fraction of points passing the gamma criteria) of 97.0 ± 1.6% on the first 182 arcs verified.

**Conclusions:**

The results of the commissioning tests and of the first period of clinical operation, resulted meeting specifications and having good margins respect to tolerances. UNIQUE was put into operation for all delivery techniques; in particular, as shown by the pre-treatment quality assurance results, it enabled accurate and safe delivery of RapidArc plans.

## Background

During 2009, a new single-energy linear accelerator for radiotherapy was introduced in clinical operation by Varian Medical System (Palo Alto, CA, USA). This new linac, called UNIQUE™ (UNIQUE in the following), was an evolution of the previous series of low-energy linacs. It incorporated new treatment modalities like Volumetric Modulated Arc Therapy according to the RapidArc^® ^method as well as advances in imaging modalities. UNIQUE also improved gantry mechanical control to allow safe operation of the advanced delivery modes. The world's first installation of UNIQUE took place at the Oncology Institute of Southern Switzerland and the machine started clinical treatments in June 2010.

Purpose of the present report was to summarise commissioning data in terms of main mechanical features as well as beam characteristics. Secondly, the results of the RapidArc commissioning on UNIQUE were presented as well as an overview of the technical aspects of the first clinical treatments. Several protocols and publications exist describing and recommending standardised procedures for beam data commissioning as well as publications on quality assurance procedures (among these, AAPM [1] or ESTRO [2] codes of practice), on analysis of results from mono or multi institutional investigations [3] and on accuracy and precision levels required in radiation therapy in general [4]. The present report, was based on recommendation from the Swiss Society of Radiobiology and Medical Physics [5] and were tailored to the specific commissioning needs to characterise a delivery system into the Eclipse treatment planning system adopted at author's institute.

## Methods

UNIQUE linac was designed to generate and deliver a single photon beam of nominal energy of 6MV with a maximum dose rate of 600 (or 400 MU/minute depending on the version), and was developed with a vertical standing wave linac, without bending magnet and steering coils. RF power generation was realised by a conventional magnetron. It was equipped with a Millennium multileaf collimator (MLC) with either 120 leaves (with 0.5 cm resolution at isocentre in the inner 20 cm and 1.0 cm resolution in the outer 20 cm) or with 80 leaves (1.0 cm resolution over the entire 40 cm of maximum field size). The couch top was derived from high energy linacs and adapted for image guidance and rotational therapy (the so-called Exact-IGRT couch top). Mechanical and Enhanced Dynamic Wedges were implemented on this new delivery platform as in other conventional Varian linacs. Mega Voltage Imaging was guaranteed by the amorphous silicon electronic portal imager PortalVision aS1000 (with pixel size of 0.392 mm) or aS1000/2 (with half resolution) operated by the so-called ExactArm, a robotic positioning arm using an active control and position correction system that compensates for gravitational and mechanical undue movements even during rotation. Patient anti-collision safety was implemented by means of a laser-based system (LaserGuard). Optional Image-guided patient repositioning was facilitated through 2D-2D MV image matching (Portal Vision Advanced Imaging (PVAI) application) and by automatic remote treatment couch movement managed by the image review application without the necessity to enter the room for couch operation.

Operational limits for asymmetric jaws were -2 cm overtravel for x jaws and -10 cm for y jaws; similarly, all other mechanical were implemented identical to other existing delivery Clinac platforms from Varian.

Concerning RapidArc implementation on UNIQUE, gantry rotation was controlled in the first generation of machines, by a slipping clutch system. The dose rate control of the UNIQUE accelerator was uses a principle schematically summarised as follows. The gun pulse trigger is always in coincidence with the magnetron pulse; the dose rate is varied by changing the magnetron pulse repetition frequency (PRF). The PRF frequency varies between 50 - 400 pulses/sec depending on the dose rate (up to 600 MU/min).

Every 50 ms, the control system of UNIQUE, compares, in dynamic treatments, the number of cumulative MU (resolution of 0.01 MU) delivered versus prescribed and takes it into account for calculation of the PRF for the next dose rate servo cycle.

### A.UNIQUE Commissioning, Anisotropic Analytical Algorithm configuration and periodic quality assurance measurements

To determine the radiation beam characteristics and to commission the Anisotropic Analytical Algorithm [6,7] used for patients dose calculation and to assess the stability of the machine performance over time, the following tests were performed and reported here.

i) Isocentre determination. A conventional star film shot procedure was performed with X-Omat V Kodak films. The specification for the isocentre sphere diameters are 2 mm. The test was repeated for different gantry, collimator and couch angle settings.

ii) Output factors. Output factors were measured for squared and rectangular fields in water at 10 cm depth and data were compared against performed calculations. Field sizes ranged from 3 × 3 to 40 × 40 cm^2^. Machine calibration was performed at isocentre at 10 cm depth for a field size of 10 × 10 cm^2^.

iii) Output stability as a function of dose rate (called MU stability) and linearity between output and MU (called MU linearity) were assessed from periodic quality assurance measurements in the range respectively from 100 to 600 MU/minute and from 5 to 300 MU. MU stability was expressed as the ratio of dose measured at a given dose rate to the reference at 300 MU/min delivery. MU linearity was expressed as the ratio of dose measurement per MU (dose/MU) at given MU to the reference 100 MU delivery.

iv) Depth doses and beam profiles in principal x and y axes were measured for a variety of square fields with the same range as at ii)..

v) Similarly to what performed for open fields, also fields modified by Mechanical and Enhanced Dynamic Wedges were investigated in terms of profiles, depth doses, output factors and wedge transmission factors.

Commissioning beam data measurements were performed in water with ion chambers: 0.125 cm^3 ^(Semiflex, PTW) for profiles and depth doses and output factors or 0.6 cm^3 ^(Farmer, Nuclear Enterprise) for absolute dose calibration. Source to phantom distance SSD was set to 90 cm for all measurements. Depth dose curves (PDD) were normalised to d_max _and profiles were normalised at the beam's central axis. A field size of 10 × 10 cm^2 ^was used to determine d_max_. Results of periodic quality assurance measurements of beam characteristics, including beam energy check, were reported in this summary, too. These were obtained by means of the portal dosimetry method GLAaS [8] as implemented in the commercial EPIQA software (Epidos s.r.o., Bratislava, Slovak Republic). For beam profiles analysis, field symmetry was defined as the maximum ratio between symmetric points within the flattened region (80% of the field size): max(D(x)/D(-x)) and expressed in percentage. Homogeneity was defined within the flattened region as (D_max_-D_min_)/(D_max_+D_min_) and similarly expressed in percentage. Field size was defined at 50% beam profile intensity. Tolerances were derived from Swiss regulations on quality assurance on linear accelerators for medical usage [5].

Beam data measured for machine commissioning, were compared against calculation performed in the Eclipse Treatment Planning System for the Anisotropic Analytical Algorithm AAA version 10.0.25 with a grid size of 2.5 mm. Details on the beam processing for AAA can be found in Fogliata et al [6]. In summary, the AAA configuration phase consisted in the optimisation of parameters and calculation kernels against the measured beam data. The optimisation is performed using objective functions including the gamma index of Low [9]. As an output of the AAA beam configuration phase in Eclipse, plots of the gamma index after optimisation are provided by Eclipse and reported here for depth doses, before and after d_max_, and for profiles in the flattened region, within the field edge and outside the field edge.

For some of the parameters, a direct comparison against either published [6,8], or institutional data for the 6MV beam generated by the high energy Clinac iX available at authors institute was provided to appraise performance of the UNIQUE beam delivery system in the absence of other published references.

### B.Imager isocenter accuracy and 2D/2D match and couch shift accuracy

The imager isocenter accuracy QA test evaluated whether the digital graticule generated by the PVAI application coincided with the treatment isocenter. The so-called marker-block phantom (a cubic phantom with one fiducial radiopaque marker at the center) was aligned on the couch with the treatment isocenter using the wall lasers. MV images at different gantry angles were acquired and analyzed measuring the distance between the center of the marker and the digital graticule inside the PVAI application (step 1 of the test).

To test the accuracy of the 2D-2D match procedure, a set of 2 orthogonal images was acquired after a manual pre-defined shift in the 3 directions of the center of the phantom: the 2D-2D match was performed to re-align the phantom, checking the proposed shift respect the expected values (step 2 of the test).

The remote couch shift was applied according to the previous match, and new images were acquired to test the couch shift accuracy (step 3 of the test). This procedure was derived from methods published by Yoo et al [10]. Weekly checks were executed at 90° and 180°, monthly frequency included also 0° and 270° but were not reported here.

### C.Rotational Stability

To assess overall accuracy and relevance of the gantry sag and imager position (ideally corrected by the arm active control of the Portal Vision system) during rotation, in view of RapidArc commissioning and quality control, tests were performed by measuring the displacement of the center of a narrow field (0.4 × 0.4 cm^2^) from its expected nominal position at 0,0 cm coordinates (in the imager coordinates system) during an entire arc executed either clock or counter-clock-wise [11]. Measurements were performed with the PortalVision. Comparison with similar measurements on an high energy linear accelerator (Clinac iX), implementing the same arm active control system, were provided for reference.

### D. RapidArc commissioning and medium term (1 year) machine performances

RapidArc (details about the principles and the algorithms can be found in Cozzi et al [12]) commissioning tests were performed according to the procedures described in the seminal work of Ling et al [13]. These tests were performed on the UNIQUE to assess the accuracy of the machine in generating uniform dose delivery with various combinations of dose rate, gantry speed and leaf speed variations during rotational delivery. Tolerance on the acceptable deviation of each dose band generated with a given combination of the above parameters from the baseline (defined as average of all the dose bands) was set to 2%. Results were provided for repeated series of measurements during the first year of UNIQUE operation. Comparison with corresponding measurements on a high energy linac (Clinac iX) were provided for reference. Data were measured by means of portal dose images [11] and analysed by means of the automatic tool implemented in the Epiqa software.

RapidArc delivery with the UNIQUE was also assessed by investigating the machine dynamic status recorded every 50 msec by the linac control system. These records were saved in the format of dynalog files where each actual dynamic parameter was stored in association to the corresponding expected parameter from delivery steering instructions. Data were recorded and analysed for each MLC leaf position, for the accumulated dose and for the gantry angle. Results were reported for a set of 12 clinical cases from our library of RapidArc plans delivered on the UNIQUE and, for comparison, on the Clinac iX unit.

Quality RapidArc delivery was also assessed at dosimetric level. For reproducibility, the same clinical plan was delivered with a biweekly periodicity while each patient treated with RapidArc on the UNIQUE underwent standard pre-treatment quality assurance measurements. Numerical analysis was performed calculating the 2D gamma of Low [9] maps from the comparison of calculated and delivered dose distributions at d_max _according to the GLAaS method [11] and scoring the Gamma Agreement Index GAI with Distance to Agreement threshold set to 3 mm and Dose Difference threshold set to 3%. Results from clinical patients included also a limited number of cases treated with fixed gantry IMRT, and data were compared with the corresponding results from other Varian linear accelerators available at authors institute.

## Results and discussion

### A. UNIQUE Commissioning, Anisotropic Analytical Algorithm configuration and periodic quality assurance measurements

#### i. Isocenter determination

**Figure 1:**
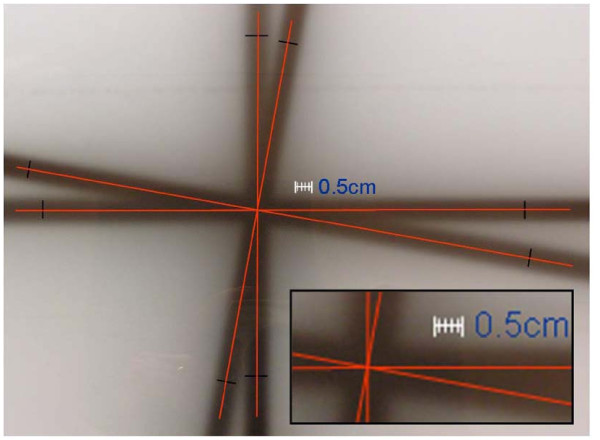
**Standard star-shot test**. for radiation isocenter determination with film: measured radius for gantry radiation isocenter sphere resulted <0.1 mm

Figure 1 represented the result of the isocenter radius determination by means of one standard star shot test. In all conditions of gantry couch and collimator settings the diameter of the sphere resulted smaller than 0.1 cm while the machine specifications required it to be <0.2 cm.

#### ii. Output factors

**Figure 2:**
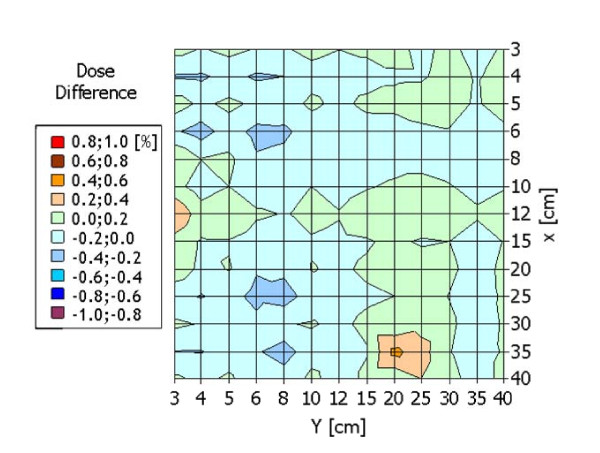
**Dose accuracy for open fields as function of field size**. (10 cm depth, SDD = 90 cm): percentage difference of calculated respect to measured dose, for 100 MU.

Figure 2 summarized the results of the output factor agreement between doses calculated in the TPS and the corresponding measurements for fixed monitor units (100 MU) in reference conditions(i.e. SSD = 90.0 cm, depth = 10.0 cm). The entire map falls within ±0.6%. The mean value is 0.0 ± 0.1%.

#### iii. Routine beam output check, MU stability as a function of dose rate and MU linearity

**Figure 3:**
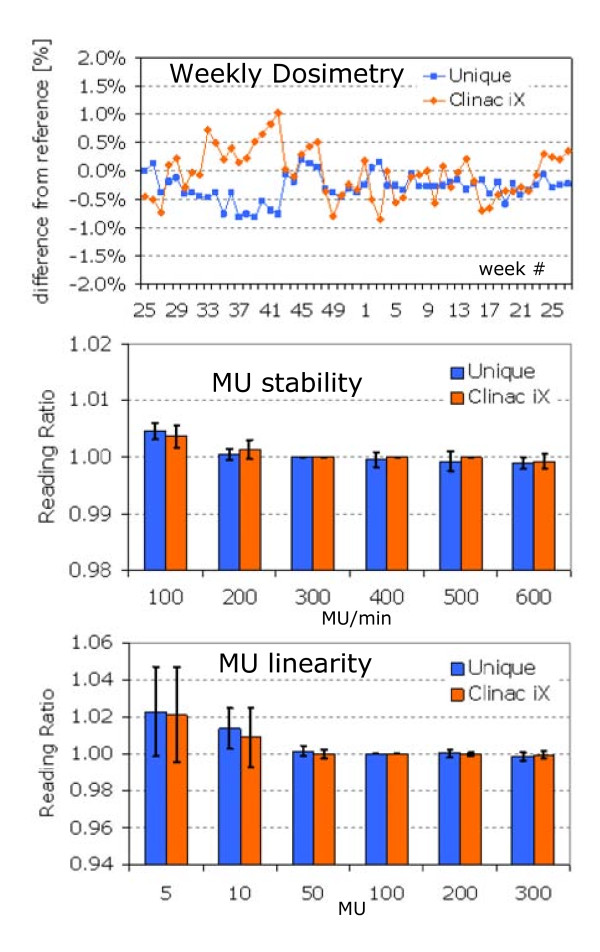
**Stability results over one year period for UNIQUE and Clinac iX**. Error bars refer to one standard deviation. A) Output (weekly check): percentage dose deviation from reference. At week n.42, machine output was re-tuned for both machines according to institutional protocols. B) MU stability (monthly check): ion chamber reading ratio for a fixed number of MU (100) between delivery at a fixed dose rate (100, 200, 300, 400, 500, 600 MU/min) and reference reading at 300 MU/min. C) MU linearity (monthly check): ratio between ion chamber reading at fixed number of MU (5, 10, 50, 100, 200, 300 MU) with respect to the same for reference of 100 MU. Measurements are relative to a fixed dose rate of 300 MU/min.

Figure 3 showed the results of the routine beam output tests performed weekly over a period of 1 year. The first graph summarized the percentage dose difference from the baseline for the reference field at 100 MU. Tolerance was ±2% while results fall all within ±1% and typically within ±0.5%. In table 1, a summary of the machine output periodic control is presented with the observed range and tolerances. The data are representative of one year period of machine operations. For direct comparison, the corresponding results for the 6MV beam generated by the Clinac iX of the institute are presented, too. MU stability and linearity results were summarized in the second and third graphs of Figure 3. MU stability with dose rate was assessed and results were within 0.5% of the reference for all dose rates; MU linearity resulted on average within ±2% below 10MU and with negligible deviations for higher values.

**Table 1:** Summary of the results of the periodic radiation beam quality assurance measurements.

	Unique		Clinac iX	
***Output ***(% difference from ref.) Tolerance: <2%	-0.3 ± 0.2% [-0.8,+0.2]		-0.1 ± 0.4% [-0.9,+1.0]	
***Energy***: Tolerance:<2%				
%diff. ratio @5.6 cm/dmax	0.0 ± 0.0 [-0.1,0.1]		0.1 ± 0.1 [-0.2,0.6]	
%diff. ratio @7.6 cm/dmax	-0.1 ± 0.1 [-0.3, 0.1]		-0.1 ± 0.1 [-0.3, 0.4]	
%diff. ratio @11 cm/dmax	-0.1 ± 0.1 [-0.3, 0.1]		-0.0 ± 0.2 [-0.3, 0.6]	
***EDW _WF ***(% difference from ref.) Tolerance: <2%	0.0 ± 0.2 [-0.1,0.1]		-0.0 ± 0.3 [-0.1,0.5]	
	
	***X dir.***	***Y dir.***	***X dir.***	***Y dir.***
	
***Field Size ***[cm] 10 × 10 cm_2_, d_max _Tolerance: <2 mm	10.03 ± 0.05(ref.10.02) [10.00, 10.14]	10.06 ± 0.06(ref.10.07) [9.99, 10.13]	10.10 ± 0.03(ref.10.02) [10.08, 10.11]	9.99 ± 0.02(ref.10.04) [9.94, 10.05]
***Field Size ***[cm] 20 × 20 cm_2_, d_max _Tolerance: <3%	20.13 ± 0.01(ref.20.13) [20.10, 20.14]	20.14 ± 0.02(ref.20.13) [20.11, 20.18]	20.21 ± 0.01(ref.20.21) [20.18, 20.24]	20.09 ± 0.02(ref.20.02) [20.02, 20.10]
***Flatness ***[%] 10 × 10 cm_2_, d_max_: Tolerance: <3%	0.8 ± 0.04 (ref.0.7) [0.7, 0.9]	0.9 ± 0.04 (ref.0.9) [0.8, 0.9]	1.2 ± 0.09 (ref.1.1) [1.0, 1.5]	0.8 ± 0.05 (ref.0.9) [0.8,1.0]
***Flatness ***[%] 20 × 20 cm_2_, d_max_: Tolerance: <3%	1.5 ± 0.04 (ref.1.5) [1.4, 1.6]	2.0 ± 0.06 (ref.1.8) [1.9, 2.1]	1.1 ± 0.12 (ref.1.0) [0.9, 1.4]	1.6 ± 0.10ref.1.7) [1.4,1.8]
***Symmetry ***[%] 10 × 10 cm_2_, d_max_: Tolerance: <103%	100.6 ± 0.2 (ref.100.6) [100.4, 100.9]	100.3 ± 0.2(ref.100.4) [100.1, 100.7]	100.5 ± 0.2 (ref.100.3) [100.3, 101.1]	100.5 ± 0.2 (ref.100.3) [100.2, 101.1]
***Symmetry ***[%]20 × 20 cm_2_, d_max_: Tolerance: <103%	101.1 ± 0.1(ref.101.3) [101.0, 101.2]	100.4 ± 0.2 (ref.100.3) [100.2, 100.7]	100.6 ± 0.2 (ref.100.4) [100.1, 101.1]	101.4 ± 0.3 (ref.101.7) [100.5, 101.8]

Measurements were performed by means of the GLAaS method (except output, where a 0.6 cm^3 ^ion chamber was used) on the UNIQUE linac and, as comparison, on the Clinac iX linac operational at authors institute. Results derived from weekly controls over a period of one year and included stability of: dose output, beam quality, EDW wedge factor, field size, beam homogeneity and symmetry. Measurement settings are reported in the first column. Mean results were shown together with their standard deviation and range (in square brackets) for UNIQUE and Clinac iX in the second and third columns. Reference baseline was reported within normal brackets

#### iv. Depth Doses and beam profiles

**Figure 4:**
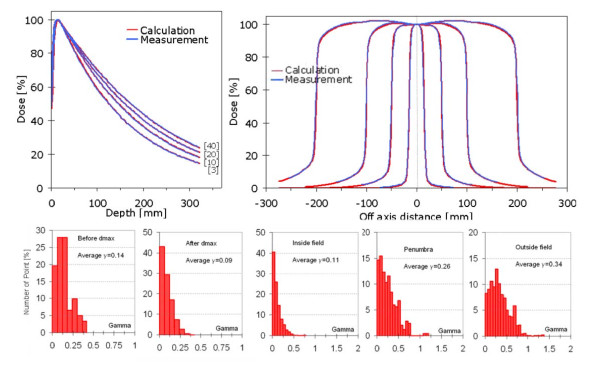
**Measured and calculated open fields**. (10 cm depth, SDD = 90 cm); calculated data refer to AAA algorithm version 10.0.25. First row: examples of profiles and DD curves; second row: gamma analysis [1%,1 mm] on all data after beam processing phase.

Figure 4 showed depth dose curves and profiles in the X direction for the fields 3 × 3, 10 × 10, 20 × 20 and 40 × 40 cm^2^. The graph reported the measured data and the corresponding curves computed from the TPS after AAA algorithm configuration. The beam quality resulted in J10/J20 = 1.740 and TPR20/10 = 0.667 (Clinac iX, 6MV, respectively 1.732 and 0.673). The histogram summarized the results of the gamma analysis during AAA processing for the five different regions described in the methods. For comparison, corresponding mean gamma values for a Clinac 6EX previously installed at the authors institute were compatible with the current: 0.17, 0.09, 0.12, 0,20 and 0.17 respectively with similar negligible fraction of points with gamma greater than 1.

Part of Table 1 summarized the results of periodic quality assurance control for field size, profile homogeneity and symmetry in the X and Y directions, and beam energy. The energy check is reported as the ratio between dose measured at different depths in solid water with respect to the corresponding value at d_max_. As can be seen, all the findings are within tolerance, the observed range was quite limited and there was a full compatibility of results with data from high energy linac.

#### v. Mechanical and Enhanced Dynamic wedges

**Figure 5:**
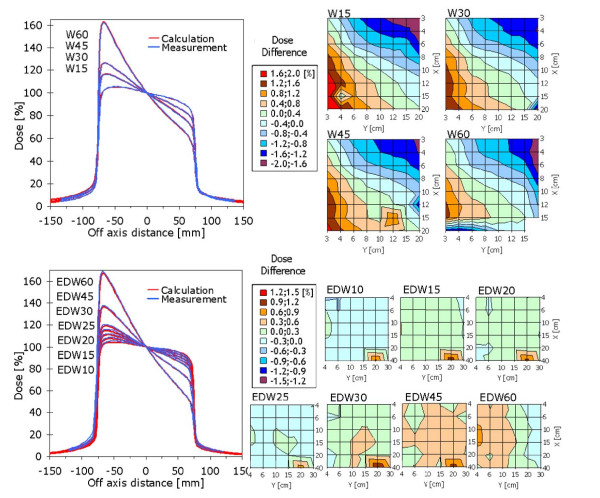
**Wedges results **: profiles and dose accuracy as function of field size, respectively A) Hard Wedges and B) EDW. Dose accuracy is defined as: percentage difference of calculated dose respect measured dose, with fixed MU, at 10 cm depth, SDD = 90 cm.

Figure 5 showed examples of mechanical and Enhanced Dynamic Wedge profiles for a 15 × 15 cm^2 ^field from measurements (acquired for both wedge types in the water phantom, with PTW LA48 linear array for the EDW case) and AAA calculations after algorithm commissioning. The dose difference maps showed, as a function of the x and y field side, the maximum percentage difference between measurement and calculation for fixed MU (100 MU). No deviations greater than ±2% were observed for all field sizes and wedges. Average deviations per mechanical wedge were: W15 -0.1 ± 1.0%, W30 -0.1 ± 0.9%, W45 -0.1 ± 0.9%, W60 -0.4 ± 0.8%. Enhanced Dynamic Wedge resulted in a much smaller range of deviations with typical ranges within ±0.6%. Average deviations were: EDW10 -0.1 ± 0.2%, EDW15 0.2 ± 0.2%, EDW20 0.1 ± 0.2%, EDW25 -0.1 ± 0.2%, EDW30 0.2 ± 0.2%, EDW45 0.4 ± 0.2%, EDW60 0.2 ± 0.3%. In table 1, the deviation from reference of Wedge Factors for 20 × 20 cm^2 ^EDW fields in the two directions In and Out, as measured with the GLAaS portal dosimetry for weekly quality assurance protocols, was reported averaged over all wedge angles and resulted compatible with 0%.

### B. Imager isocenter accuracy and 2D/2D match and couch shift accuracy

**Figure 6:**
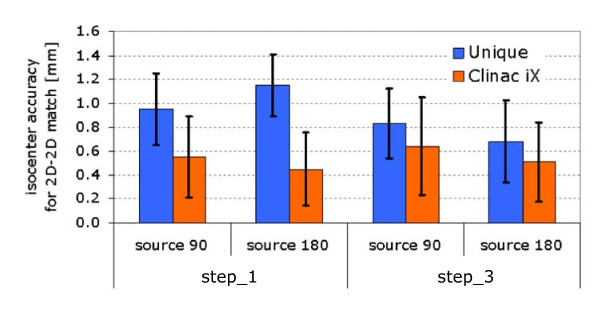
**Image guidance on UNIQUE**. Verification of 2D-2D image matching with weekly quality assurance procedure: average results (and standard deviation) over one year period. For comparison results for the same procedure on Clinac iX are presented although these latter refer to images acquired with the MV and kV systems, for steps 1 and 3 of the protocol.

Figure 6 showed the results of imager isocenter (step 1) and couch shift (step 3) accuracy over a six-month period on weekly basis for both UNIQUE and Clinac iX. The images were acquired at 180° and 90°, i.e. the standard positions used for 2D imaging in our institute induced by the most common start position of the first arc for RapidArc treatment (i.e. 179° as internal rule). To notice that, for the Clinac iX, the 2 images were acquired respectively with MV and kV detectors to minimize gantry movements. The average results were respectively for UNIQUE and Clinac iX 1.0 ± 0.3 and 0.5 ± 0.3 mm at 90°, 1.2 ± 0.3 and 0.4 ± 0.3 mm at 180° for step 1, 0.8 ± 0.3 and 0.6 ± 0.4 mm at 90°, 0.7 ± 0.3 and 0.5 ± 0.4 mm at 180° for step 3, always lower than acceptability criteria set at 1.5 mm. About the disagreement between the remote couch shift obtained from 2D-2D match and the expected shift of 1 cm (step 2), was always less than 1 mm.

### C. Rotational Stability

**Figure 7:**
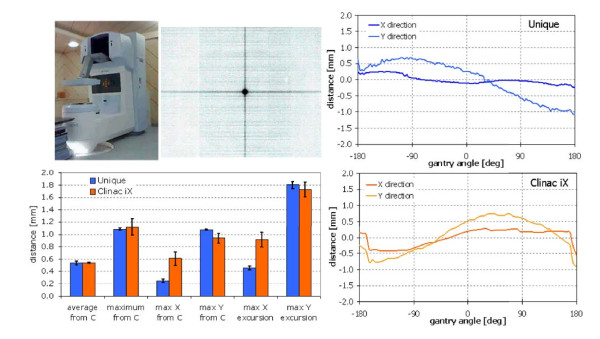
**Comprehensive test of the stability of the imaging center**. (including PV mechanical stability and gantry sag motion) from monthly quality assurance procedures. Test was performed acquiring portal images in continuous (cine) mode with a full gantry rotation. The graph reported an example of the actual distance of the center C of the small radiation field from the physical image center in the X and Y directions as a function of the gantry angle; the histogram showed the average deviations observed over a test period of one year as well as the maximum distance, the maximum deviation in X and in Y directions and also the maximum excursion of the deviations; error bars are expressed as 1 standard deviation.

Figure 7 showed the results of the gantry rotational stability tests. A small field (0.4 × 0.4 cm^2^) was acquired in cine mode with the portal imager and the relative movement in x and y directions of its center of mass was plot against gantry angle. The histogram showed the results of monthly tests over a period of 1 year. As it can be seen, the total residual motion due to gantry sag and portal imager displacement due to gravity not compensated by the active arm control system is on average <0.6 mm with a maximum deviation from the nominal center <1.2 mm and absolute maximum excursion in the y direction <1.8 mm.

### D. RapidArc commissioning and medium term (1 year) machine performances

**Figure 8:**
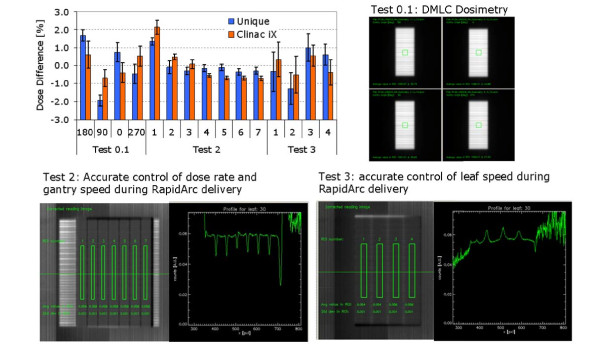
**Summary of the RapidArc commissioning tests**. (according to Ling et al [13]) performed as monthly checks over a period of one year. The plot showed the dose output difference between readings in each uniform band from the average values for: test 0.1 (a dynamic IMRT field with a 0.5 cm slit at 4 different gantry positions); test 2 (seven different combinations of dose rate and gantry speed during a RapidArc delivery) and test 3 (four different combinations of MLC speed and dose rate during a RapidArc delivery). Recommended tolerance was ±2% for all tests. Images for tests 2 and 3 are corrected for the beam profile, rationing the band and the open field acquisitions.

**Figure 9:**
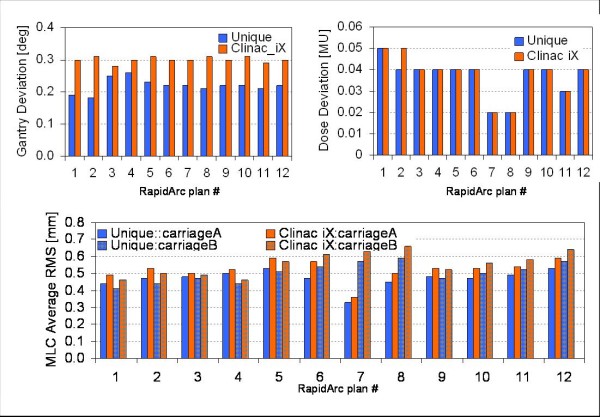
**Summary of the Dynalog Files analysis**. for 12 test cases from real clinical patients delivered on both UNIQUE and Clinac iX linacs. Plot showed the average deviations from reference or expected values during arc delivery of: gantry angle, accumulated MU and RMS of MLC positions.

Figure 8 reported the results of the monthly tests performed according to the referenced study of Ling et al [13]. Test 0.1 referred to fixed gantry deliveries, while tests 2 and 3 referred to rotational deliveries, with different combinations of gantry speed, dose rate and MLC speed. Each test aimed to generate uniform dose delivery in bands as shown in the figure. Tolerance of 2% for the maximum deviation in each band from the baseline defined as the average over all bands was required and on average achieved in all cases for UNIQUE, even in the challenging first band of test 2, where gantry inertia was shown to be sometimes critical also in previous experience. The tests performed at commissioning and periodically over 1 year, demonstrated that the rotational control system of UNIQUE is accurate and precise for RapidArc delivery and allowed for immediate clinical implementation of this technique. Delivery parameters were investigated for plans of 12 patients delivered on both UNIQUE and Clinac iX by means of dynalog files analysis. Figure 9 summarizes for each of these test cases the average deviation from planned/expected positions of the gantry, of the MU and of the MLC. In all cases both machines showed i) very small inter patient variability and ii) very small absolute deviations from theoretical reference. Interestingly, the gantry deviation plot showed better results on UNIQUE than on Clinac iX. This systematic effect was linked to different tightening of the chain or clutch systems but did not induced measurable dosimetric effects.

Quality assurance of RapidArc delivery included also i) delivery of standardized clinical test cases for RapidArc and also for IMRT to prove global machine stability and ii) pre-treatment verification of clinical plans for all patients as described in the methods. Table 2 summarized the results of these measurements. GAI for the constancy tests resulted fully equivalent with reference historical data from other machine available at institute, further showing reliability of the UNIQUE. At the time of submission, 152 patients (192plans, 348 arcs) were treated for RapidArc on UNIQUE and for these cases, GAI resulted of 97.3 ± 1.6% with a complete overlap with historical results from a larger group of 606 patients (797 plans, 1186 arcs) treated on a period of 31 months with RapidArc on Clinac iX at the institute.

**Table 2:** Summary of the stability control and of the pre-treatment patients quality assurance results for RapidArc and IMRT treatments.

		Unique	Clinac iX
GAI [%] constancy on a pre-treatment QA case (1 year data with a periodicity of 2 weeks)	RapidArc case	98.5 ± 1.1 [96.7, 99.6]	99.0 ± 0.3 [98.3, 99.4]
	IMRT case	99.4 ± 0.1 [99.2, 99.2]	99.0 ± 0.4 [98.2, 99.3]
Clinical pre-treatment RapidArc QA	GAI [%]	97.3 ± 1.6 [92.4, 99.9]	97.4 ± 1.8 [91.5, 99.9]
	Number of arcs (plans)	348 (192) [12 months]	1186 (797) [31 months]

Beside UNIQUE summary, a comparison with similar results from the Clinac iX operational at the institute is shown. Data are expressed as Gamma Agreement Index GAI with Distance to Agreement and Dose Difference thresholds set to 3 mm and 3%. Measurements and calculations were performed according to the GLAaS method by means of portal dosimetry.

## Conclusions

A new-generation of low-energy linear accelerator, UNIQUE, was recently introduced in the clinical arena (at the moment with the exclusion of USA, Canada and Japan) by Varian Medical Systems. The results of the commissioning tests and of the first period of clinical operation of this new delivery system were presented in this report for beam characterisation and modelling into the treatment planning system, periodic quality assurance tests and RapidArc operations. In all areas, UNIQUE resulted meeting specifications and having good margins respect to tolerances, and was put into operation for all delivery techniques. In particular, as shown by the pre-treatment quality assurance results, it enabled accurate delivery of RapidArc plans and this ended in the interruption of clinical application of IMRT at our institute having replaced the entire fixed gantry IMRT programme with RapidArc now enabled on all delivery systems of our institute.

## Competing interests

Dr. L. Cozzi acts as Scientific Advisor to Varian Medical Systems and is Head of Research and Technological Development to Oncology Institute of Southern Switzerland, IOSI, Bellinzona.

## Authors' contributions

AF and LC coordinated the study. Data acquisition and data analysis were done by AC, EV, GN, AF. The manuscript was prepared by LC and GN. All authors read and approved the final manuscript.
